# From childhood socio-economic position to adult educational level – do health behaviours in adolescence matter? A longitudinal study

**DOI:** 10.1186/1471-2458-13-711

**Published:** 2013-08-02

**Authors:** Leena Kristiina Koivusilta, Patrick West, Vesa Markus Antero Saaristo, Tapio Nummi, Arja Hannele Rimpelä

**Affiliations:** 1 University Consortium of Seinäjoki, Seinäjoki Technology and Innovation Centre,, University of Tampere, School of Health Sciences, Kampusranta 9C, Seinäjoki FIN-60320, Finland; 2 Department of Social Research, University of Turku, Turku, Finland; 3 MRC Social & Public Health Sciences Unit, Glasgow, Great Britain; 4 National Institute for Health and Welfare, Finn-Medi 3, Tampere, Finland; 5 School of Health Sciences, University of Tampere, Tampere, Finland; 6 Department of Adolescent Psychiatry, Tampere University Hospital, Tampere, Finland

**Keywords:** Adolescence, Health behaviours, School career, Educational level, Structural equation modeling

## Abstract

**Background:**

Our interest was in how health behaviours in early and late adolescence are related to educational level in adulthood. The main focus was in the interplay between school career and health behaviours in adolescence. Our conceptual model included school career and health-compromising (HCB) and health-enhancing (HEB) behaviours as well as family background. Two hypotheses were tested: 1) the primary role of school career in shaping educational level in adulthood (an unsuccessful school career in adolescence leads to HCB and not adopting HEB and to low educational level in adulthood); 2) the primary role of health behaviours (HCB and not adopting HEB in adolescence leads to a school career with low education in adulthood).

**Methods:**

Mailed surveys to 12 to18 year-old Finns in 1981–1991 (N=15,167, response rate 82%) were individually linked with the Register of Completed Education and Degrees (28 to 32-year-olds). We applied structural equation modeling to study relations of latent variables (family SEP, family structure, school career, HCB, HEB) in adolescence, to the educational level in adulthood.

**Results:**

Standardized regression coefficients between school career and health behaviours were equally strong whether the direction was from school career to HEB (0.21-0.28 for 12–14 years; 0.38-0.40 for 16–18 years) or from HEB to school career (0.21-0.22; 0.28-0.29); and correspondingly from school career to HCB (0.23-0.31; 0.31-0.32) or from HCB to school career (0.20-0.24; 0.22-0.22). The effect of family background on adult level of education operated mainly through school career. Only a weak pathway which did not go through school career was observed from behaviours to adult education.

**Conclusions:**

Both hypotheses fitted the data showing a strong mutual interaction of school achievement and adoption of HCB and HEB in early and late adolescence. Both hypotheses acknowledged the crucial role of family background. The pathway from health behaviours in adolescence to adult education runs through school career. The interplay between behaviours and educational pathways in adolescence is suggested as one of the mechanisms leading to health inequalities in adulthood.

## Background

Education fundamentally shapes an individual’s social position and living conditions [1] and is strongly related to health [1]; people with lower levels have poorer health than those with higher levels [2,3]. The school career of a child is influenced by the socio-economic characteristics of the family; school achievement and selection of educational routes after compulsory education varying according to parents’ education, socioeconomic position and family structure [4]. Children of highly educated white-collar parents are more likely to reach the highest level of education than other groups [5], and compared with children of continuously married parents, those of divorced parents have lower academic achievement and associated risk factors, e.g., conduct problems, psychological adjustment, self-concept, and social relations [6,7]. The importance of family characteristics for adolescents’ health behaviours has been consistently demonstrated over time [8,9].

Adolescence is the phase in an individual’s life course when many health-compromising and health-enhancing behaviours are adopted. It is also a stage during which important decisions are made regarding the extent and direction of education – usually based and shaped by achievements in school. Children and adolescents following a health-compromising behavioural pattern perform, on average, less well at school than those with health-enhancing behavioural patterns and attain lower educational levels in adulthood [10-12]. A recent review suggested that some health behaviours causally impact educational outcomes [13].

Pathways leading to different educational levels in adulthood operate mainly through family socioeconomic position and family structure, an adolescent’s school career consisting of achievements and selection of different educational tracks. While health behaviours in adolescence influence health later in life, and while the educational level of an individual also impacts health, the interplay of these factors suggests a more complex mechanism that may lead to health differences between educational groups in adulthood. Studies into the origin of health inequality are often focused either on independent predictors of health or educational level or on the clustering of health behaviours among young people into healthy or unhealthy patterns [10,14-16]. Health-compromising behaviors, such as smoking or excessive alcohol use, are more prevalent among those with a lower educational level; and health-enhancing behaviors, such as tooth brushing and physical activity, are more prevalent among those with higher levels [17-21]. Yet, the roles and interactions of behavioural and sociodemographic factors in the formation of educational processes are less well investigated [22].

**Figure 1:**
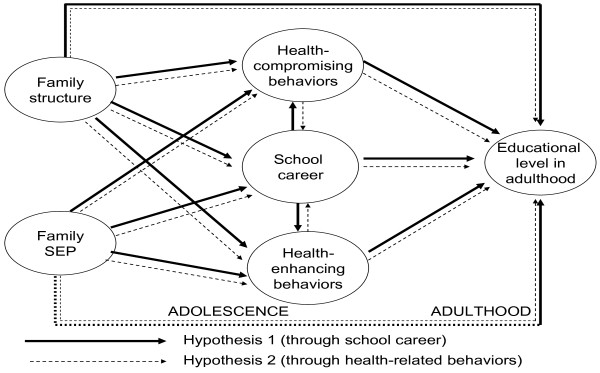
The conceptual model of the study: the hypothesized pathways from family context through school career/health behaviors to adult education (Hypotheses 1 and 2).

The conceptual model of the present study is shown in Figure 1. Over and above the relationships between family background, school career and adult educational level, the core of our interest lies in the middle of the figure where the intertwining of the school career and health behaviours is represented. These processes are presented in the form of two hypotheses. According to Hypothesis 1 (solid lines), an unsuccessful school career would lead to a greater probability of adopting health-compromising behaviours and of not adopting health-enhancing behaviours, while a successful school career would lead to a behavioural pattern favourable to health. Consequently, adolescents with favourable health-related behavioural patterns would end up with a higher educational level in adulthood, those with unfavourable patterns with lower educational levels. As school achievement is strongly influenced by family background, this pathway mediates the impact of home on adult educational levels.

According to Hypothesis 2 (dotted lines), no engagement in health-compromising behaviours and adoption of health-enhancing behaviours in adolescence would impact a school career leading to higher educational levels in adulthood, while engagement in health-compromising behaviours and no engagement in health-enhancing behaviours could impact educational tracks leading to lower educational levels. For this hypothesis, the impact of family background on a school career is hypothesised to go via the health-related behaviours on the choice of educational track. In addition to these pathways, a pathway from family background to educational level in adulthood is possible. Hypotheses 1 and 2 were tested in this study.

## Methods

### Data and variables

Baseline data were obtained from the Adolescent Health and Lifestyle Surveys of 1981, 1985, 1987, 1989 and 1991. Nationally representative samples of 12-, 14-, 16-, and 18-year-old Finns born on certain days in July, June and August were drawn each study year from the Population Register Center. The response rate was 82% (N=15,167) and, by age-sex groups, 83% in 12-and 14-year-old boys (N=2502) and 91% in girls (N=2704), and 72% in 16- and 18-year-olds boys (N=4680) and 86% in girls (N=5281). A self-administered questionnaire was sent in February, followed by two re-inquiries to non-respondents. The variables in this study were based on similar questions in each survey.

Follow-up data, the highest attained educational level, were obtained from the Register of Completed Education and Degrees containing information on every resident in Finland. Statistics Finland performed the data linkage according to a contract specifying the rights and duties of both parties. The study protocol was approved by the Data Protection Ombudsman.

Follow-up ended 31 December, 2001, when participants reached the ages of 28 to 38 and most had completed their education. The variable *educational level in adulthood* was based on the person’s highest educational attainment [23]: higher degree-level tertiary or doctorate (16+ years in education), lower degree-level tertiary (14–16 years), lowest tertiary (13–14 years), upper secondary (11–12 years), basic (includes lower secondary) education (9–10 years, or no completed education. Each participant had a value in the Register. We excluded 125 (0.8%) baseline respondents who had died during the follow-up.

The baseline variables were categorized from most “favourable” to most “unfavourable” in terms of socioeconomic position, school career and health behaviours. The repeatability of the variables had been previously tested and shown to be good [24]. The following six constructs (five exogenous and one endogenous) formed the latent variables (measurement part) in our statistical models.

Family socioeconomic position (SEP) was described by the *father’s or guardian’s occupation* (Statistics Finland, 1989: upper white-collar employee, lower white-collar employee or farmer, blue-collar employee) and *father’s or guardian’s educational level*: high (over 12 years), middle (9–12 years), and low (at most 9 years). The correlations between father’s/guardian’s occupation and education (family socioeconomic position) varied between the age-sex groups from 0.74 to 0.79.

Family structure, measured by *family type,* was categorized as nuclear (living with both parents) and other.

School career in adolescence was measured by *School attainment* at ages 12 and 14, based on the end of term school report. Adolescents were asked whether it was much better than the class average, slightly better, average, slightly below, much below average. *Educational track* was used for 16- and 18-year-olds, some of whom had finished school. According to the type of school and school attainment respondents were classified into seven categories presumed to predict their education in adulthood, the first category having the highest probability of reaching a high level of education in adulthood, the seventh the lowest probability: upper secondary school (1) with above-average school achievement, (2) with average achievement, (3) with below-average achievement; vocational or other schools (4) with above-average school achievement, (5) with average school achievement, (6) with below-average school achievement; (7) not attending school.

Health-compromising behaviours were *smoking*: never tried, smoked once, smoked 2 to 50 times, smoked over 50 times, smokes less than 10 times daily, smokes at least 10 times daily; and *alcohol drinking style*: abstinence, occasional drinking, recurring drinking (drinks alcohol at least once a month), recurring drunkenness (drinks until really drunk at least once a month). The correlations between smoking and alcohol drinking style varied from 0.56 to 0.67 in the age/sex subgroups.

Health-enhancing behaviours. *Intensity of weekly physical activity* summarized information from five questions which measured frequency of physical activity: participation in sports and physical activity organized by 1) sports clubs, 2) school or workplace (physical training lessons excluded), 3) other associations/clubs, 4) practised alone or with friends/family members, and 5) the extent of getting out of breath or sweating during physical activity. The derived categories were: very active vigorous activity, vigorous activity, occasional vigorous activity, light activity, and no activity. *Frequency of brushing teeth* was categorized as: several times a day, once a day, about 4 to 5 times a week, about 2 to 3 times a week, at most once a week, never. The correlations between weekly physical activity and brushing teeth varied from 0.10 to 0.19 in the age-sex subgroups.

Educational level in adulthood was measured by one indicator variable only. This endogenous factor is our main variable of interest.

### Statistical analysis

Structural equation modeling with the Lisrel 8.71 program [25] was used to study how five latent variables characterising the baseline situation in adolescence were related with our main variable of interest, *educational level in adulthood*. These latent (exogenous) variables were *family structure*, *family socioeconomic position*, *school career, health-compromising behaviours* and *health-enhancing behaviours in adolescence*. Polychoric correlation coefficients of pairwise present cases from the PRELIS program [25,26] were used to quantify the associations between the measured variables in the four age/sex subgroups. The models were fitted by the method of weighted least squares with polychoric correlation coefficients.

Three models were fitted separately in age-sex groups. First, the models (basic models) including associations between family background, school career and educational level were fitted. This was followed by fitting the models relating to Hypothesis 1 and Hypothesis 2 (Figure 1). Then, the same model was fitted including only statistically significant associations at a 5% risk level (t-statistic smaller than −1.96 or bigger than +1.96). Standardized regression coefficients (range 0–1) for the associations are presented. The fit of these models was evaluated by means of root mean square error (RMSEA), an adequate fit of the model indicated by RMSEA<0.08 [27].

## Results

**Figure 2:**
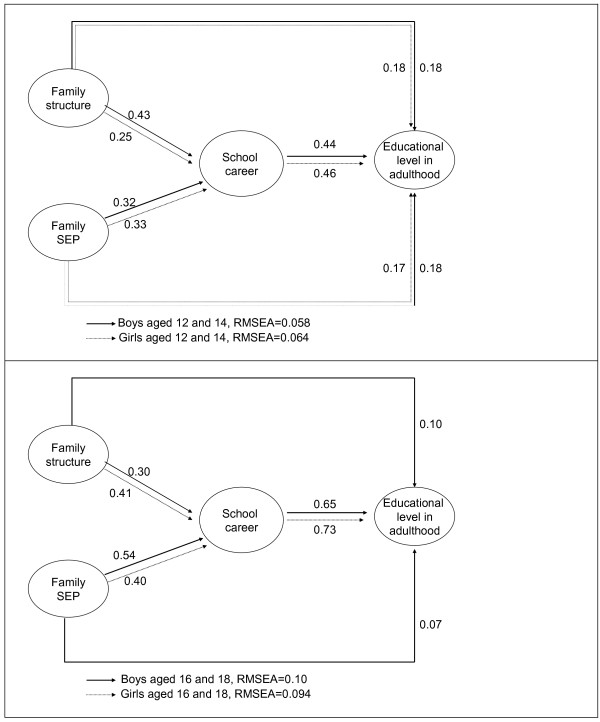
**Standardized regression estimates for statistically significant (*t *test) in the fitted structural equation models: Basic model for boys and girls aged 12 and 14 years (top panel) and 16 and 18 years (lower panel) at the baseline.**

We first tested the model without health behaviours (the basic model) in order to estimate the strength of the relationships between family background, school career and adult educational level. The significant associations in Figure 2 show a strong pathway from family background (SEP and family structure) to adult education through school career in all age-sex groups and, except for 16–18 year-old girls, a weak association from family background to adult education.

**Figure 3:**
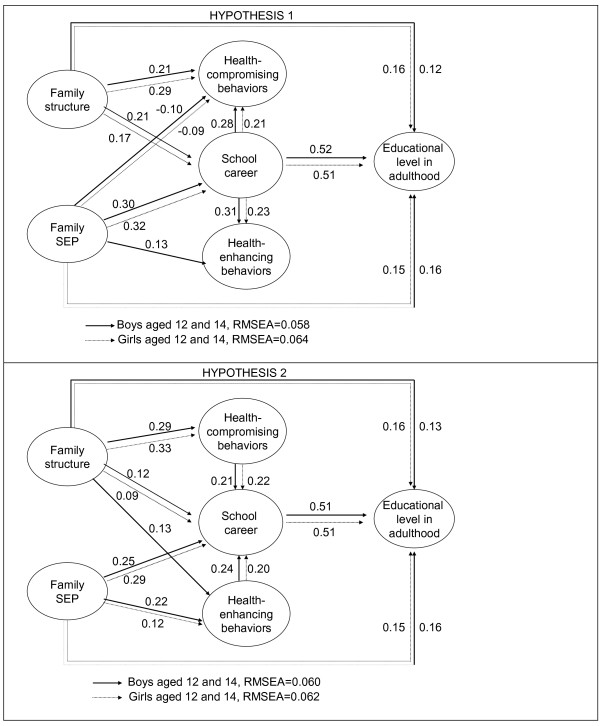
**Standardized regression estimates for statistically significant (*t *test) associations in the fitted structural equation models among boys and girls aged 12 and 14 years at the baseline: Hypothesis 1 (top panel) and Hypothesis 2 (lower panel).**

**Figure 4:**
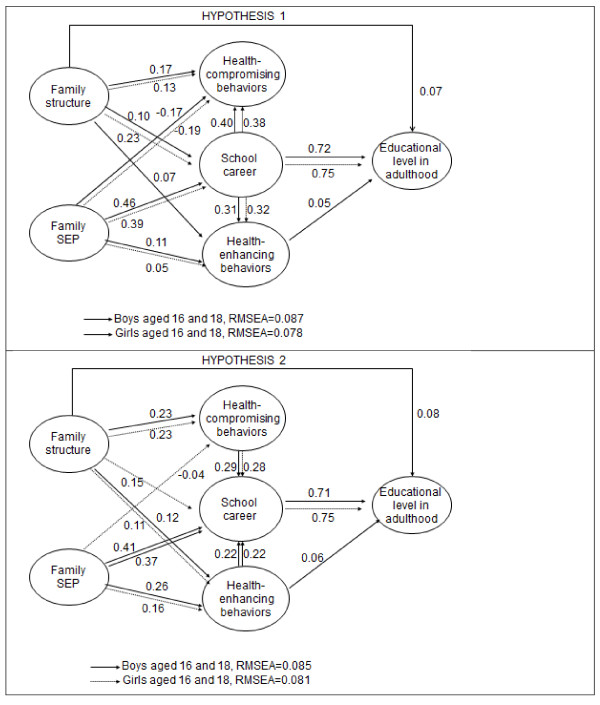
**Standardized regression estimates for statistically significant (*t *test) associations in the fitted structural equation models among boys and girls aged 16 and 18 years at the baseline: Hypothesis 1 (top panel) and Hypothesis 2 (lower panel).**

Figures 3 and 4 present the standardized regression estimates for the statistically significant associations in the models for both hypotheses.

### 12-14-year-olds

#### Hypothesis 1

There were strong associations between school career and both health-compromising and health-enhancing behaviours; an unfavourable school career more often led to health-compromising behaviours and less often to health-enhancing ones (Figure 3, top). There were no independent connections from either type of behaviours to education in adulthood. A non-nuclear family structure signified more involvement with health-compromising behaviours, but was not associated with health-enhancing behaviours. Higher family SEP had a weak negative impact on health-compromising behaviours in both sexes and a positive impact on health-enhancing behaviours among boys. The impact of family background variables on adult educational level remained unchanged compared to the model in Figure 2, but the path from family structure to educational career became weaker, and that from school career to adult educational level somewhat stronger.

#### Hypothesis 2

This model (Figure 3, lower) showed a remarkably similar set of connections to those relating to Hypothesis 1, the coefficients between school career and the two health behaviours being of similar magnitude and, in this case, of opposite direction. As before, the pathway from family structure and family SEP to adult educational level went predominantly through school career and no pathways from the health behaviours to education in adulthood were observed.

### 16-18-year-olds

#### Hypothesis 1

In common with younger adolescents, there were strong connections from school career to health-compromising and health-enhancing behaviours, an unfavourable school career more often leading to health-compromising behaviours and less often to health-enhancing ones (Figure 4, top). However, a weak connection was observed between health enhancing behaviours and adult educational level, but only in boys. Boys and girls from a non-nuclear family structure were also more likely to be involved in health-compromising behaviours and less involved in health-enhancing behaviours. High family SEP had a weak negative impact on health-compromising behaviours and a positive impact on health-enhancing behaviours. A connection from family structure to adult educational level was observed among boys only, but no connections from family SEP to adult educational level were apparent.

#### Hypothesis 2

Compared to the model of Hypothesis 1, the connections between health-compromising behaviours and school career were weaker (Figure 4, lower). In addition, while school career and adult educational level remained strongly related, the connection between family SEP and health-enhancing behaviours increased, and that between family structure and health-enhancing behaviours was now observed for both sexes. The connection between family structure and health-compromising behaviours was also observed in both sexes. In this model, family SEP was also related to school career in both sexes. As in the model of Hypothesis 1, the only connection from health behaviours to educational level in adulthood was a weak one from health-enhancing behaviours among boys. Finally, there was a weak path from family structure to adult educational level in boys, a nuclear family background positively impacting on higher education.

In both models, associations from school career to adult educational level were stronger among older compared to younger adolescents. The fit of all models, according to the criteria, was adequate.

## Discussion

We tested two hypotheses on how health behaviours in early and late adolescence are related to educational level in adulthood. The main interest was the interplay between school career and health behaviours in adolescence, a time when several health behaviours are established and educational paths selected. The first hypothesis suggested the primary role of school career: an unsuccessful school career would lead to a greater probability of adopting health-compromising behaviours and of not adopting health-enhancing behaviours, followed by a lower educational level in adulthood. The second hypothesis suggested the primary role of health behaviours: engagement in health-compromising behaviours and not adopting health-enhancing behaviours leading to a school career that in turn led to lower educational level in adulthood. Both hypotheses acknowledged the crucial role of family background.

We could not show that one of the hypotheses is essentially better than the other which suggests a strong mutual interaction of school career and the adoption of health compromising and health enhancing behaviours in adolescence. Just as school career has a crucial role in determining the adoption of health-compromising and health-enhancing behaviours as well as adult educational level, so the reverse is true; behaviours impact on school career. This is seen during the compulsory schooling age (12 to 14 year olds) as well as after that when children have been recruited to different schools or not continuing school (16–18 year olds).

No pathways from health behaviours to educational level in adulthood were observed in girls or younger boys, their effect operating through school career. Health behaviours, thus, strongly contribute to the educational level in adulthood, but only through their interrelationship with school career in adolescence. Among older boys, however, there was a weak pathway from health-enhancing behaviours to educational level in adulthood which most likely is related to the high intensity of physical activity that has been shown to predict a higher level of education in adulthood [11].

In both early and late adolescence, the strongest pathway from family background to educational level in adulthood operates through school career, much weaker paths being observed for the younger adolescents only. This highlights the significance of processes operating before the important decisions about continuation of education after compulsory education are taken.

The pathways from family background through school career to educational level are consistent with those of studies from other Western countries showing that home background continues to play a decisive role in educational choices and educational success [28,29]. Various reasons have been given for this, ranging from educational values prevailing in families of different social strata [30] to economic inequality between families [5]. The importance of family structure was also confirmed, adolescents living with both parents having a greater probability of good educational attainment [31] The role of family structure was important also in the adoption of health-compromising behaviours while family SEP was more prominent in the adoption of health-enhancing behaviours.

Family background, school career and health-compromising and health-enhancing behaviours are a mixture of processes leading to different educational levels in adulthood. From a health point of view, this is suggested as one of the mechanisms by which health inequalities in adulthood are established, and which are evident in the risk factor profiles of people with high and low education.

### Strengths and limitations of the study

We used large, nationwide samples which were followed up to age 28 to 38 years through the national register, a reliable source of the highest education of Finnish residents [32]. At the surveys, response rates were good. The boys’ lower participation led to a slightly higher proportion of females compared with the population of the same age. Respondents represented the Finnish population according to their educational level [23].

Statistical analysis was based on the polychoric correlations within each latent variable. Correlations were strong between the variables for family SEP, and health-compromising behaviours (smoking, alcohol) which has been shown elsewhere, too [14,33]. Correlations were weak for the variables measuring health-enhancing behaviours (tooth brushing, physical activity); however, both behaviors are known to predict high education [10,11]. The classification of behaviours into health-compromising and health-enhancing dimensions is not without problems [34,35] but follow-up was feasible only for those health behaviour variables collected in each survey.

The reliability and validity of self-reported behavioural measures is good in adolescence [36,37]. The repeatability of our questions has been shown to be good [24]. In health-related studies, non-response is associated with low parental education, poor school performance, and unhealthy behaviours [38]. Even if the associations between these three factors differed among the non-respondents, it is unlikely that the effect would be large enough to affect the results. School career and health related behaviours in adolescence were measured simultaneously in the same surveys. Thus, the study doesn’t allow inferences about causality and the directions of the associations between health behaviours and school career. Also, a better understanding of their mutual relationship would have been achieved if more than one measurement during adolescence had been available.

## Conclusions

In both early and late adolescence, there is a strong mutual interaction of school achievement and adoption of health compromising and health enhancing behaviours. Both hypotheses acknowledged the crucial role of family background. The pathway from health behaviours in adolescence to adult level of educational runs through school career. The interplay between behaviours and educational pathways in adolescence is suggested as one of the mechanisms leading to health inequalities in adulthood.

## Competing interests

The authors declared that they have no competing interests.

## Authors’ contributions

AR and LK planned the study design. LK, PW and AR participated in the writing of the manuscript. LK and VS performed the statistical analyses under the supervision of TN. All authors read and approved the final manuscript.

## Pre-publication history

The pre-publication history for this paper can be accessed here:

http://www.biomedcentral.com/1471-2458/13/711/prepub
